# Genomic Insights From Natural History Collections Reveal Cryptic Speciation in Coral Guard Crabs (Family: *Trapeziidae*)

**DOI:** 10.1002/ece3.70960

**Published:** 2025-02-19

**Authors:** Kenzie Pollard, Carlos Leiva, Heloise Rouzé, Sarah Lemer

**Affiliations:** ^1^ Marine Laboratory University of Guam Mangilao Guam USA; ^2^ Department of Evolution and Ecology University of California Davis California USA; ^3^ Museum of Nature Hamburg Leibniz Institute for the Analysis of Biodiversity Change, Center for Molecular Biodiversity Research Hamburg Germany

**Keywords:** coral reefs, Crustacea, cryptic species, exosymbiont, genome, Guam, Marquesas Islands, Museomics

## Abstract

Mutualistic relationships such as the one between *Trapezia* crabs and coral colonies are common in reef organisms and play a crucial role in coral resilience and resistance to climate‐induced stressor, yet very little is known about the taxonomic diversity and evolutionary history of the species involved. Despite being essential actors of coral reefs and threatened by the ongoing degradation of their habitat, little genetic information is available for *Trapezia* crabs, including the exact number of species and their relationships. To overcome this limitation, we sampled Natural History Collections, an important and underutilized source of genomic data. We used a novel approach optimized for degraded DNA to generate high‐quality genomic data from a combination of 166 museum tissues and freshly collected samples and recovered a strongly supported phylogeny of the *Trapezia* genus, clarifying species relationships of a majority of taxa and suggesting the potential division of *Trapezia* into two genera. We then focused on the most widespread species *T. bidentata* and identified four distinct genetic clusters, suggesting high divergence and cryptic speciation in the Indian Ocean and the Marquesas Islands. Populations of the Central and West Pacific showed signs of admixture across a heterogeneous seascape, attributing to a potentially long pelagic dispersal phase and expansive gene pool. Our results highlight the need to further explore the genetic diversity within other *Trapezia* species and other coral‐associated organisms, as they are likely to exhibit more complex genetic patterns than previously understood.

## Graphical abstract

We explored the evolutionary history of Trapezia crabs, key coral mutualists, using genomic data from museum and freshly collected samples. We recovered a well‐supported phylogeny of the Trapezia genus, clarifying species relationships and supporting the division of Trapezia into two genera. Detailed analyses of the widespread species *T. bidentata* identified four distinct genetic clusters and two cryptic species in the Indian Ocean and the Marquesas Islands, while populations in the Central and West Pacific showed signs of admixture, indicating extensive gene flow. These findings underscore the need for further investigation into the genetic diversity of Trapezia and other coral‐associated species, as they likely exhibit more complex patterns than previously known.

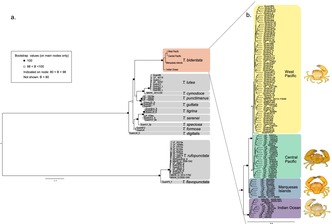

## 1 Introduction

Mutualistic relationships are common in reef organisms and enhance reef productivity via increasing coral host survival (Castro 1988; Leray et al. 2012; Rouzé et al. 2014). These mutualistic interactions involve not only symbiotic unicellular microalgae of the family Symbiodiniaceae but also various invertebrates and fish. Growing evidence indicates that these coral‐associated organisms are essential for coral resilience and resistance to climate‐induced stressors, including heat stress, ocean acidification, and increased sedimentation (summarized in Stier and Osenberg 2024a). However, due to their often elusive nature and the challenges in studying them, much of their diversity remains unknown, severely limiting our understanding of their contributions to coral health. In this study, we address this knowledge gap by focusing on a symbiosis that has been gaining recognition: the relationship between the coral guard crab genus *Trapezia* Latreille, 1826, and Pocilloporid corals (Stier and Osenberg 2024a). Found throughout the Indo‐Pacific (Castro 2000), these mutualistic crabs live in the interstitial spaces of coral colonies and assist corals by removing sediments (Stewart et al. 2006), deterring crown of thorns seastar predation (Glynn 1980; Pratchett 2001) and preventing incursion by corallivorous and mucus secreting snails (Shima et al. 2010; Stier et al. 2010). Further, *Trapezia* crabs can help mitigate stress and decrease mortality of juvenile colonies when they are most sensitive to external stressors (Stewart et al. 2006; Rouzé et al. 2014; Stier and Osenberg 2024a). The crabs feed primarily on detritus, coral mucus, and other bacteria that reside or land on the coral tissues (Castro 1976) and rely on the intricate branching of their coral host for protection from predators (Castro et al. 2004). They live in mated pairs alongside congenerics, exhibiting high levels of territoriality toward conspecifics (Huber 1985; Castro et al. 2004; Glynn 2013). Additionally, they are susceptible to elevated temperatures, often resulting in the eviction of females by male crabs during heat stress, suggesting a fragile balance between cooperation and competition (Stella et al. 2014). Female *Trapezia* will brood eggs post‐internal fertilization and release nauplius larvae into the water column as free‐swimming plankton (Gotelli et al. 1985). Because of their planktonic life phase, they have a potentially broad dispersal range; however, the length of this free‐swimming phase is unknown. While several species of *Trapezia* have been identified over the years, little is known about the diversity within each species and the structure of adjacent populations. On a broader scale, various reconstructions of the *Trapezia* phylogeny have hinted at the existence of cryptic species (i.e., low morphological but high genetic disparity) within several of the broadly dispersing clades— *T. bidentata* , *T. lutea* , *T. cymodoce* and *T. digitalis* (Huber 1985; McKeon 2010; Rouzé et al. 2017; Canizales‐Flores et al. 2020). Recognizing cryptic diversity (i.e., the presence of multiple distinct species lumped into one because of morphological similarity) is imperative to determining the vulnerability of species and the persistence of these mutualisms, as different species experience different sensitivities to changing climates (Bickford et al. 2007). For the purposes of this study, we will be focusing on *Trapezia bidentata* (Forskål, 1775), a candidate for cryptic diversity.

*Trapezia bidentata*, previously *T. ferruginea* Latreille, 1828, is believed to have a broad distribution spanning the entire Indo‐Pacific region with records of the species existing as far east as Baja California and Panama and as far west as Madagascar and the Red Sea. The species' broad distribution indicates a long‐lived lineage with high dispersal potential; however, the timing of its speciation and the length of its free‐swimming pelagic larval phase is unknown. *Trapezia bidentata* has a characteristic orange carapace and black pinchers as well as diagnostic red dots on the terminal ends of its ambulatory legs (Castro et al. 2004). It is classified as a medium‐size species within *Trapezia* (Gotelli et al. 1985) and is thus effective at deterring *Culcita novaeguineae* corallivory (McKeon and Moore 2014) and most often found inhabiting small to medium‐size *Pocillopora* coral species. A few studies have found hints of cryptic speciation (i.e., the evolutionary process resulting in morphologically similar but genetically divergent species) in *T. bidentata* from the Indian and Pacific Oceans including Rouzé et al. (2017) who analyzed two mitochondrial markers (COI and 16 s) in populations of *T. bidentata* from the Reunion Islands and New Caledonia, and McKeon (2010) who described a divergence between the Indian Ocean and Pacific using a mitochondrial COI marker and numerous sampling sites. Further, McKeon (2010) suggested distinct coloration between the two potential allopatric sister taxa. There are several known biogeographical barriers in the Indo‐Pacific that have been documented to influence species distributions over time, such as eustatic sea level changes, habitat availability, currents, and temperature and salinity gradients (Crandall et al. 2019; Keyse et al. 2018). Because *T. bidentata* spans such a broad geographic range (Castro 1997; Castro et al. 2004), the possibility that multiple species have been grouped under *T. bidentata* based on morphological similarity should be considered, as this could deceptively inflate its perceived geographic range.

Increasing our understanding of the vulnerability of these obligate exosymbionts and clarifying their phylogenetic relationship and within‐species distribution is of major importance to the health of coral reefs, especially in assessing their capacity to adapt to changing climates. However, and because they live hidden between coral branches, obtaining genomic data from *Trapezia* crabs, or for any other endangered, rare, cryptic, or hard‐to‐catch taxa, is hindered by the challenge of accessing a comprehensive collection of samples that encompass geographic range and within‐species phenotypic diversity (Blom 2021). In these situations, Natural History Collections and museum specimens in particular, can provide important sources of genetic data (Bi et al. 2013; Supple and Shapiro 2018; Lemer et al. 2019; Combosch et al. 2017). Yet, a significant constraint of museum samples lies in the fact that archival, historical, or ancient DNA is often fragmented or damaged from prolonged storage and varied preservation conditions (Twort et al. 2021; Raxworthy and Tilston Smith 2021; Ernst et al. 2022; Bi et al. 2013). Here, we addressed this challenge by applying a recently developed library preparation protocol capable of extracting high yields of genomic data from degraded archival DNA (Enoki and Takeuchi 2019; Enoki 2019; Hosoya et al. 2019). We combined over 100 museum specimens of *Trapezia* crabs with freshly collected specimens to clarify the phylogenetic relationships of elusive mutualistic coral guard crab species, test the hypothesis that *T. bidentata* represents a complex of cryptic species influenced by historical biogeographical barriers rather than one ubiquitous species, and assess its dispersal capacity throughout its geographic range.

## 2 Materials and Methods

### 2.1 Sample Collection and DNA Extraction

To clarify the phylogeny of *Trapezia*, tissue samples from 12 species were obtained either from the collections of the Florida Museum of Natural History and the Paris Museum National d'Histoire Naturelle or by sampling live specimens directly from the reefs of Guam (Special permit for scientific research License # SC‐MPA‐22‐001; Table 1 and further details in Table S1). Species included *T. lutea* , *T. guttata* , *T. serenei*, *T. rufopunctata* , *T. flavopunctata* , *T. punctimanus*, *T. speciosa* , *T. digitalis* , *T. formosa* , *T. cymodoce*, *T. tigrina*, and *T. bidentata*. To investigate the phylogeography of *T. bidentata* across the Indo‐Pacific, 68 samples were obtained from the Florida Museum of Natural History and the Paris Museum National d'Histoire Naturelle from 15 localities (Table 1 and further details in Table S1). Each specimen was photographed and subsampled for DNA extraction. To supplement museum samples, a total of 69 individuals of *T. bidentata* were collected from Guam. Sampling of live specimens in Guam was conducted using eugenol (10% ethanol, 10% clove oil, and 80% seawater) to sedate the crabs before removing them from the coral colony with metallic forceps and placing them in 50 mL falcon tubes with seawater. Falcon tubes containing crabs were stored in a chilled cooler while being transported to the University of Guam Marine Laboratory. Upon arrival at the lab, crabs were photographed live, transferred to cryovials with 95% ethanol, and stored in a −20°C freezer until extraction.

**TABLE 1:** *Trapezia* sampling information with species ID, number of samples, localities, and collection methods.

Species	Locality	Total # samples	# museum samples	# fresh samples
*Trapezia bidentata*	Society Islands, French polynesia	21	21	—
	Tuamotu Islands, French Polynesia	4	4	—
	Marquesas Islands, French Polynesia	11	11	—
	Ofu, American Samoa	2	2	—
	Kanton, Phoenix Islands	1	1	—
	Malden, North Line Islands	2	2	—
	Caroline, South Line Islands	2	2	—
	Guam, Mariana Islands	73	4	69
	Zealandia Bank, Mariana Islands	1	1	—
	Majuro, Marshall Islands	1	1	—
	Palmyra Atoll, North Line Islands	2	2	—
	Gulf of Aqaba, Saudi Arabia	5	5	—
	Papua New Guinea	1	1	—
	Reunion Island	2	2	—
	Madagascar	2	2	—
	Mayotte	2	2	—
*Trapezia rufopunctata*	Society Islands, French Polynesia	1	1	—
	Australia	1	1	—
	Taiwan	1	1	—
	American Samoa	1	1	—
	Marquesas Islands	5	5	—
	Ryukyu Islands	1	1	—
	New Caledonia	1	1	—
	Mayotte	2	2	—
	East Pacific (unspecified)	1	1	—
	Guam, Mariana Islands	5	—	5
*Trapezia lutea*	Malden, North Line Islands	2	2	—
	Guam, Mariana Islands	6	—	6
*Trapezia guttata*	Guam, Mariana Islands	3	—	3
*Trapezia serenei*	Guam, Mariana Islands	3	—	3
*Trapezia flavopunctata*	Guam, Mariana Islands	1	—	1
*Trapezia punctimanus*	Society Islands, French Polynesia	3	3	—
*Trapezia speciosa*	Guam, Mariana Islands	1	—	1
*Trapezia digitalis*	Guam, Mariana Islands	1	—	1
*Trapezia formosa*	Guam, Mariana Islands	2	—	2
*Trapezia cymodoce*	Papua New Guinea	1	1	—
*Trapezia tigrina*	Malden, Line Islands	1	1	—
	Guam, Mariana Islands	3	—	3

DNA was extracted using the GenCatch Genomic DNA Extraction kit (EPOCH Life Sciences, Missouri City, TX), following the manufacturer's protocol, and eluted in 100 μL of nuclease‐free water. DNA and residual RNA concentrations were then measured via high‐sensitivity DNA assays on a Qubit 3.0 fluorometer (Thermo Fisher Scientific Inc., Waltham, MA).

### 2.2 GRAS‐Di Library Construction, Sequencing, and Locus Assembly

A total of 180 samples from 12 *Trapezia* species were selected for Genotyping by Random Amplicon Sequencing‐Direct (GRAS‐Di, Enoki and Takeuchi 2019). GRAS‐Di is a PCR‐based genotyping by sequencing technology that facilitates genome skimming of especially degraded or low‐quality DNA samples (Hosoya et al. 2019). This is because unlike (dd)RAD‐Seq, GRAS‐Di uses random primers instead of restriction enzymes to amplify random regions of the genome, which eliminates unnecessary shearing of the DNA. Reads obtained with GRAS‐Di are filtered and analyzed identical to (dd)RAD‐Seq reads and are similarly useful for species without reference genomes. Library preparation and sequencing was outsourced to the UC Davis Genome Center.

Quality filtering, locus assembly of raw reeds, and genotyping were conducted in STACKS v2.60 (Catchen et al. 2011, 2013). Short reads were demultiplexed, adaptors were removed, and sequences were trimmed to 100 bp using process_shortreads. To optimize STACKS parameters, five random samplings of three individuals each were used to trial *M* = 1–9, as in Jeffries et al. (2016). Once *M* was set, all samples were included to trial *n* = 3–5, allowing only for SNPs present in 80% of samples (Paris et al. 2017). The remaining parameters were kept at default settings (*m* = 3, *r* = 0.5, and min‐maf = 0.05). This assessment was repeated for both the phylogeny and phylogeography datasets. To compare and assess the quality of reads obtained from fresh samples and museum specimens, the proportion of each sample in the total library and the number and proportion of reads retained in each sample after filtering were calculated with the function stacks‐dist‐extract (Rivera‐Colón and Catchen 2021). Sample coverages were obtained via the denovo.log output file (Table S2).

Two distinct datasets were prepared for the phylogeny and phylogeography analyses. For the phylogeny dataset, loci assembly parameters were set to *m* = 3, *M* = 4, and *n* = 5 to retain loci that were present in 50% of individuals (*r* = 0.5), with an average coverage of 12X. Individuals with a proportion of single nucleotide polymorphisms (SNPs) less than 20% were considered uninformative and removed with GenePop v1.2 (Raymond and Rousset 1995). The minimum minor allele frequency was set to 5%, and putative SNPs under selection (14 loci and 107 SNP) were removed with BayeScan v.2.1 (Foll and Gaggiotti 2008) using default parameters (5000 iterations, 20 pilot runs). A total of 164 samples represented by 3,386 loci and 11,478 SNPs were retained after filtering for this *Trapezia* phylogeny dataset (see Figure S4).

The phylogeography dataset, which initially included 133 *T. bidentata* individuals, was similarly filtered, resulting in assembly parameters set to *m* = 3, *M* = 4, and *n* = 5, retaining loci present in 50% of individuals, with an average coverage of 16X. Similarly, individuals with a proportion of SNPs less than 20% were removed and a minimum minor allele frequency was set to 5%. Additionally, putative loci under selection (602 loci and 1027 SNPs total) were identified with BayeScan v.2.1 (as above) and removed. Lastly, a single SNP per locus was selected in STACKS using a maximum‐likelihood (ML) model to minimize the effect of linkage disequilibrium. After filtering, a total of 119 samples represented by 4,484 loci and 4,481 SNPs were retained for this dataset.

### 2.3 Phylogenetic Reconstruction of *Trapezia*

Phylogenetic reconstruction of 12 *Trapezia* species was based on consensus sequences generated with the STACKS population program ‐phylip‐var. Maximum Likelihood analyses using IQTREE 1.6.12 (Kalyaanamoorthy et al. 2017; Hoang et al. 2018; Nguyen et al. 2015) were conducted with an initial model selection step, tree reconstruction, and 1500 ultrafast bootstrap replicates. The optimal model of sequence evolution selected was TVM + F + G4, according to the Bayesian Information Criterion (BIC), and the model was run with the SNP ascertainment bias correction to remove nonvariant sites from the alignment. In the absence of outgroup species in our dataset, the consensus tree was midpoint rooted in figtree v 1.4.4 (Rambaut 2018).

A single SNP per locus was selected to estimate genetic distance between all monophyletic groups and minimize the effects of linkage disequilibrium. Distances were estimated via measures of Reynold's and Nei's minimum distances calculated using the R package adegenet (Jombart 2008).

### 2.4 Population Genetics of *Trapezia bidentata*

Admixture analyses were performed in STRUCTURE 2.3.4 (Pritchard et al. 2000) using the admixture ancestry model with prior population membership and correlated allele frequencies to visualize fine‐scale genetic structure throughout the Indo‐Pacific. The analysis was conducted with 10 iterations for each *K* value from 1 to 8 with 150,000 Markov Chain Monte Carlo generations and a burnin of 25,000. The optimal *K* value was inferred in Structure Harvester (Earl and vonHoldt 2012) using the ad hoc posterior probability models of [Pr(X|*K*)] (Pritchard et al. 2000) and deltaK (Evanno et al. 2005) and visualized with PopHelper (Francis 2017). To determine whether sub‐structure was present within the major genetic clusters, subsequent STRUCTURE analyses were conducted within each of the main genetic cluster as described above.

A principal component analysis (PCA) using the phylogeography dataset was constructed with the R package ade4 and dudi.pca (Dray and Dufour 2007) with implementation of fviz_pca_ind from the factoextra package for better visualization (Kassambara and Mundt 2020). Pairwise *F*_ST_ values between each of the genetic clusters identified by the PCA and STRUCTURE analyses were estimated using the STAMPP package in R (Pembleton et al. 2013). *P*‐values were calculated at a 95% confidence interval with 1000 bootstrap iterations. Observed and expected heterozygosity (*H*_*O*_ and *H*_*S*_, respectively) and the inbreeding coefficient *F*_*IS*_ were calculated for each genetic cluster using adegenet and hierfstat (Goudet 2005) packages in R. A hierarchical analysis of molecular variance (AMOVA) was further implemented, via the poppr package in R, to detect significant variation between the genetic clusters (Kamvar et al. 2014). Lastly, an isolation by distance (IBD) analysis was conducted in dartR of the Pacific populations excluding the Marquesas Islands (Gruber et al. 2018). Additionally, STRUCTURE, Pairwise *F*_ST_ values, AMOVA, and PCA analyses were conducted independently on putative SNPs under selection to isolate and explore the effects of natural selection. Genetic distances between clusters were estimated as described above and compared to genetic distance between *Trapezia* species.

### 2.5 Morphometric Analysis of *Trapezia bidentata*

Discrepancies in carapace morphology across genetic clusters were quantitatively estimated by comparing similarly sized specimens from each genetic cluster, including 12 individuals (seven male and five female) from the Central Pacific, 12 individuals (seven male and five female) from the West Pacific, 14 individuals (seven male and seven female) from the Marquesas Islands, and 9 individuals (six male and three female) from the Indian Ocean. ImageJ was used to measure the length (from frontal region to before the abdomen) and width (between outer edge of eye sockets) of the carapace and a one‐way analysis of variance (ANOVA) was run to test for significance between clusters (Schneider et al. 2012; Sarower et al. 2021). In addition to sorting by cluster, the presence of significant differences between sexes was also tested, as sexual dimorphism is typically found in crustaceans (Rufino et al. 2004; Josileen 2011; Hajjej et al. 2016).

## 3 Results

### 3.1 Sampling Yields

**FIGURE 1:**
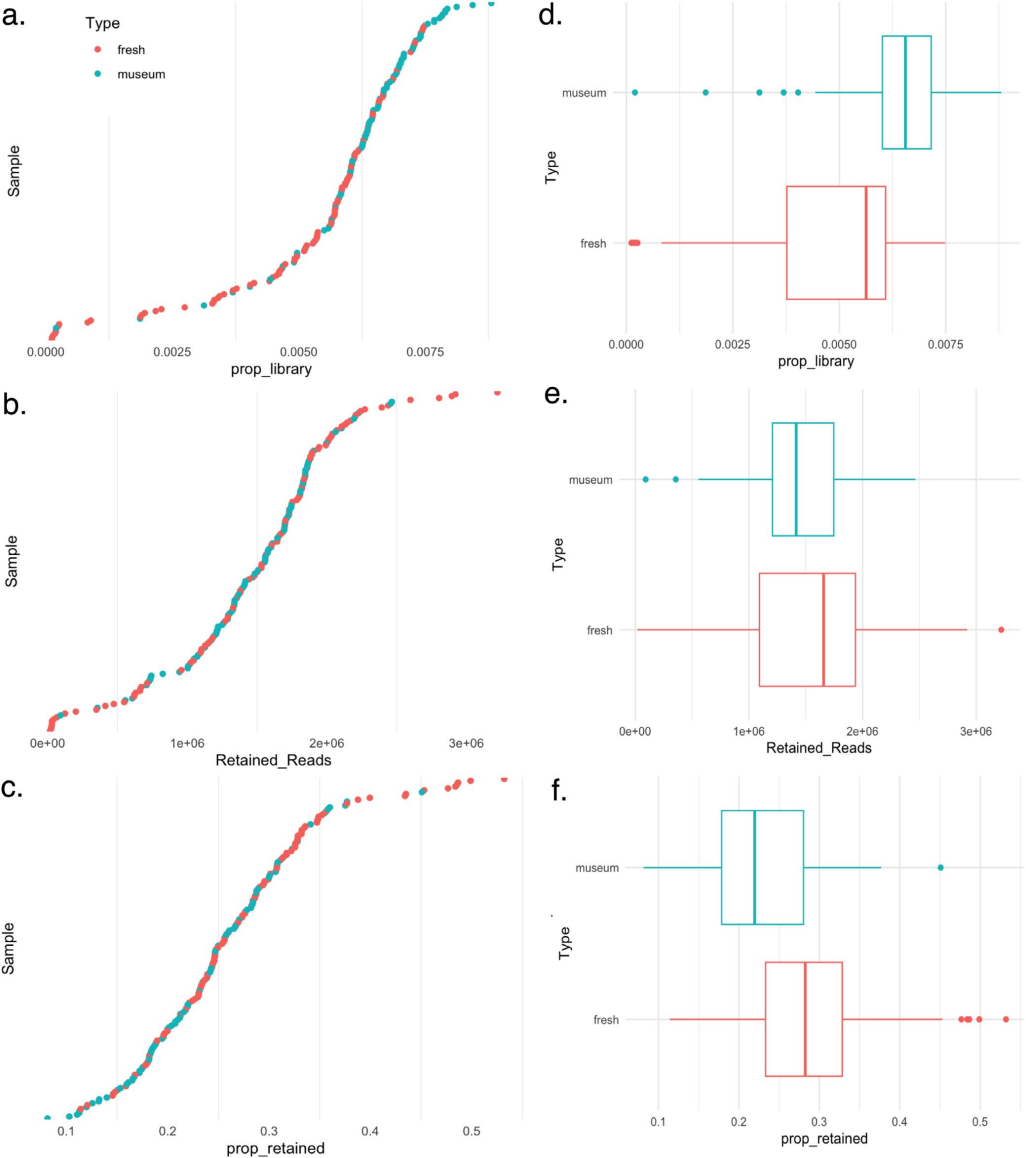
Read retention analysis comparing museum tissue and freshly collected samples. (a) Proportion of the library per sample with the corresponding boxplot (d). (b) Number of retained reads per sample with corresponding boxplot (e). (c) Proportion of retained reads after filtering with corresponding boxplot (f). Blue refers to museum samples, and red refers to fresh samples.

The range of DNA concentrations obtained from extractions conducted on museum and fresh samples varied greatly (Table S2). The DNA concentrations of museum and fresh samples were significantly different (*T*‐test: *p* = 0.005) with averages of 8.2 ± 10.6 and 14.6 ± 19 ng/μL, respectively. However, retained reads were not significantly different between museum and fresh samples (*T*‐test: *p* = 0.710), and both read types took up similar proportions of the library for raw reads (Figure 1 and Table S2). Approximately 10% of fresh samples and 2% of museum samples were filtered out due to low quality. Coverage was similar between museum and fresh samples,14.8X ± 1.5 and 13.5X ± 2.3, respectively (Figure 1 and Table S2). Further, there was no correlation between DNA concentration and the proportion of retained reads after filtering (Linear regression: *R* ^2^ = 9.45 ± 13.9, *p* = 0.498).

### 3.2 *Trapezia* Phylogeny

**FIGURE 2:**
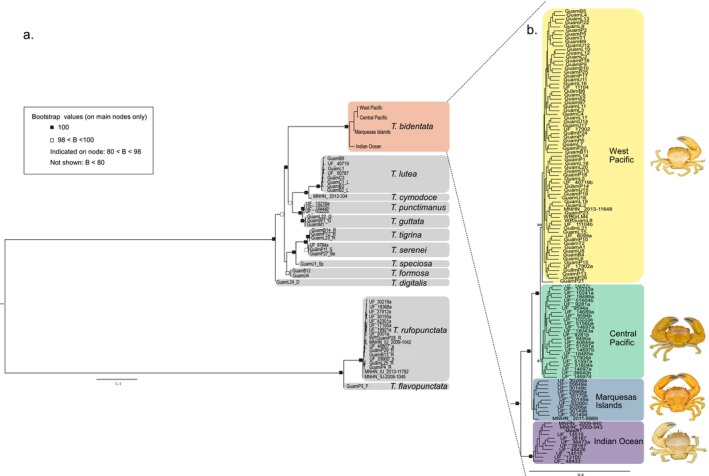
Phylogenetic tree of *Trapezia* genus (a) and *T. bidentata* (b) constructed in IQTREE. Bootstrap values of main nodes are denoted by filled squares (100% BS), open squares (98 < BS < 100), and numeric value (80 < BS < 98). Missing values indicate a BS value < 80. Examples of specimens are depicted next to their corresponding cluster.

Out of the 68 samples of *T. bidentata* collected from the museums, 9 samples were misidentified as they grouped with other species in the phylogeny (Table S1). The genus phylogeny, generated with 11,479 SNPs, displayed a well‐supported topology for all 12 *Trapezia* species (Figure 2a). The position of *T. bidentata* in relation to other *Trapezia* was clarified and recovered as a sister species to the clade containing *T. lutea* , *T. cymodoce*, *T. punctimanus*, and *T. guttata* , with full nodal support (100% BS). *Trapezia lutea* and *T. cymodoce* were placed as sister species, and so were *T. punctimanus* and *T. guttata* , with full nodal support (100% BS). Similar to previous constructions of the *Trapezia* phylogeny (Canizales‐Flores et al. 2020; McKeon 2010 unpublished work), *T. rufopunctata* and *T. flavopunctata* form a very divergent sister clade to all other species (100% BS). Finally, *T. digitalis* was recovered as sister to all *Trapezia* species, with the exception of *T. rufopunctata* and *T. flavopunctata* , with full nodal support (100% BS; Figure 2a). Within *T. bidentata* , samples clustered in two main monophyletic clades with full nodal support (100% BS), a Pacific and an Indian Ocean clade (Figure 2b). Within the Pacific clade, individuals from the Marquesas islands formed a distinct but poorly supported clade sister to all other Pacific individuals (37% BS). The remaining Pacific individuals clustered in two sister clades: a Micronesian‐Melanesian clade (94% BS; Mariana islands, Marshall Islands, Phoenix islands, North Line Islands and Papua New Guinea), hereafter named the Western Pacific clade; and a Polynesian clade [83% BS; French Polynesia (excluding Marquesas), South Line Islands and American Samoa], hereafter named the Central Pacific clade (Figure 2b).

Measurements of genetic distances between species (Nei's D and Reynold's D; Table 2) showed that the greatest genetic distances are found between *T. rufopunctata* and *T. flavopunctata* with the rest of the species (average 1.62 ± 0.06 and 0.60 ± 0.04 for Nei's and Reynold's D, respectively). The smallest distances are observed between *T. tigrina* and *T*. *serenei* (0.12 and 0.41 for Nei's and Reynold's D, respectively) as well as between *T. rufopunctata* and *T. flavopunctata* (0.27 for both distances). Significantly, Reynold's distances between *T. serenei* and *T. tigrina* (0.41) and *T. guttata* and *T. punctimanus* (0.45) were similar to or lower than the distance between the *T. bidentata* Indian Ocean cluster and the rest of *T. bidentata* (average 0.59 ± 0.01).

**TABLE 2:** Genetic distance between species (including *T. bidentata* clusters). Nei's D is in the bottom diagonal (blue) and Reynold's D is in the top diagonal (green). Darker colors indicate a greater pairwise distance.

	*T. serenei*	*T. lutea*	*T. cymodoce*	*T. punctimanus*	*T. guttata*	*T. tigrina*	*T. speciosa*	*T. formosa*	*T. digitalis*	*T. rufopunctata*	*T. flavopunctata*	*T. bidentata—Indian Ocean*	*T. bidentata—West Pacific*	*T. bidentata—Marquesas Is*.	*T. bidentata—Central Pacific*
*T. serenei*	—	0.66	0.60	0.61	0.62	0.41	0.53	0.57	0.57	0.64	0.61	0.77	0.76	0.76	0.76
*T. lutea*	0.51	—	0.55	0.62	0.63	0.67	0.62	0.61	0.60	0.67	0.64	0.79	0.78	0.78	0.78
*T. cymodoce*	0.63	0.35	—	0.57	0.59	0.62	0.56	0.55	0.53	0.58	0.55	0.73	0.72	0.72	0.72
*T. punctimanus*	0.49	0.42	0.54	—	0.45	0.63	0.58	0.57	0.56	0.63	0.60	0.75	0.74	0.74	0.74
*T. guttata*	0.50	0.42	0.56	0.18	—	0.64	0.59	0.58	0.57	0.63	0.61	0.76	0.74	0.75	0.74
*T. tigrina*	0.12	0.49	0.61	0.50	0.50	—	0.55	0.58	0.58	0.65	0.63	0.79	0.78	0.78	0.78
*T. speciosa*	0.34	0.51	0.63	0.51	0.52	0.34	—	0.53	0.53	0.59	0.56	0.73	0.72	0.72	0.72
*T. formosa*	0.42	0.47	0.58	0.45	0.48	0.42	0.43	—	0.51	0.59	0.57	0.73	0.72	0.72	0.72
*T. digitalis*	0.56	0.58	0.68	0.57	0.58	0.55	0.59	0.50	—	0.55	0.52	0.72	0.71	0.71	0.71
*T. rufopunctata*	1.60	1.61	1.67	1.65	1.59	1.59	1.55	1.50	1.53	—	0.27	0.74	0.74	0.74	0.74
*T. flavopunctata*	1.68	1.70	1.70	1.73	1.63	1.67	1.61	1.56	1.60	0.27	—	0.72	0.71	0.71	0.71
*T. bidentata—*Indian Ocean	0.62	0.56	0.68	0.54	0.54	0.60	0.62	0.58	0.72	1.80	1.83	—	0.59	0.58	0.60
*T. bidentata—*West Pacific	0.63	0.56	0.69	0.54	0.54	0.60	0.62	0.59	0.72	1.83	1.85	0.07	—	0.37	0.26
*T. bidentata—*Marquesas I*s*.	0.62	0.56	0.68	0.53	0.53	0.60	0.62	0.58	0.71	1.82	1.84	0.07	0.02	—	0.37
*T. bidentata—*Central *Pacific*	0.62	0.56	0.69	0.53	0.53	0.60	0.61	0.58	0.72	1.82	1.84	0.08	0.01	0.02	—

### 3.3 Phylogeography and Population Genetics of *T. bidentata*

**FIGURE 3:**
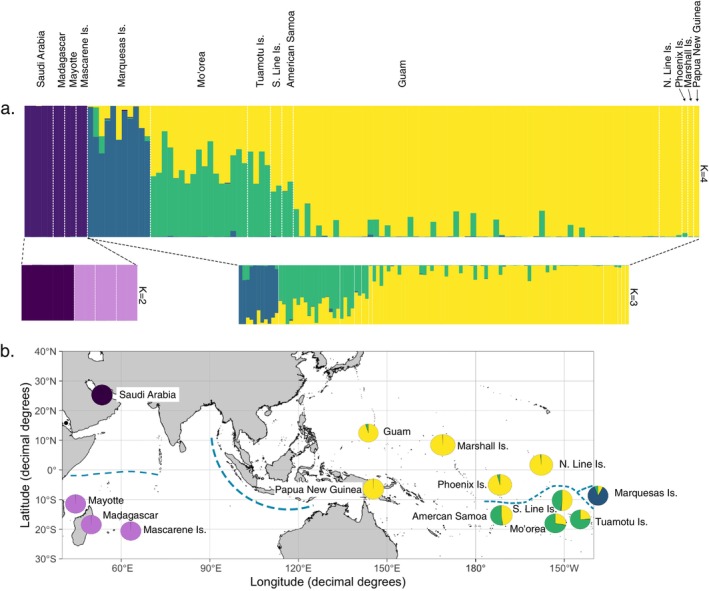
STRUCTURE analysis of *T. bidentata* for *K* = 4 with the sub‐structure of the Indian Ocean cluster for *K* = 2, below (a). (b) Map of localities with pie charts indicating admixture proportions. Each genetic clusters is represented by the same color throughout the figure (purple = Indian Ocean, yellow = West Pacific, green = Central Pacific, blue = Marquesas Islands). Dotted blue lines indicate suggested genetic breaks.

**FIGURE 4:**
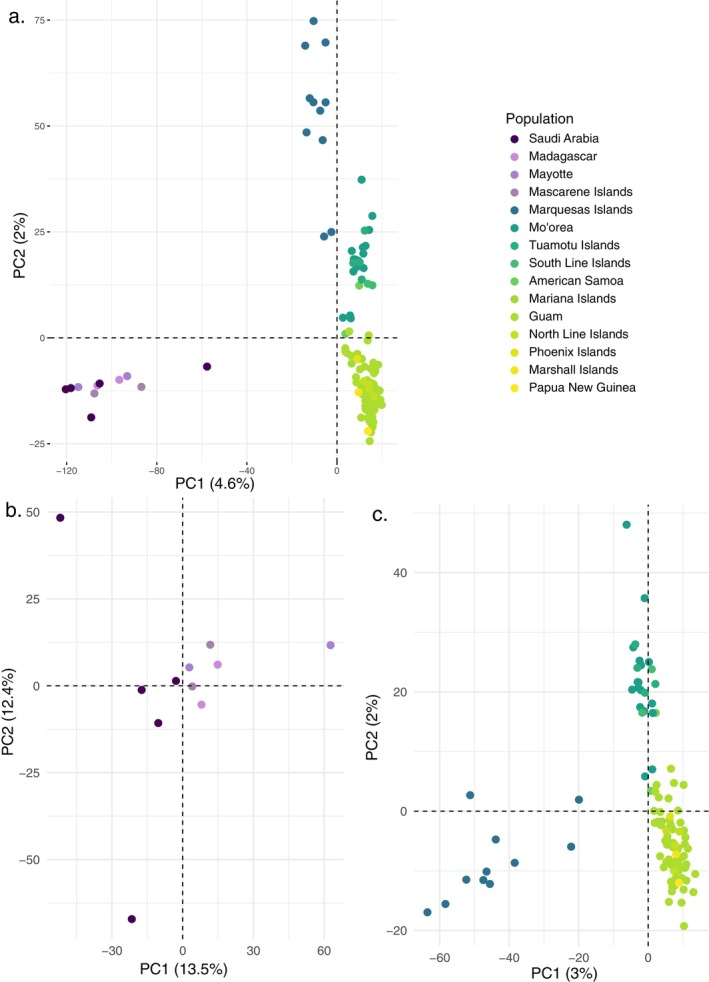
Principle component analyses of (a) all samples of *T. bidentata* , (b) *T. bidentata* in the Indian Ocean, and (c) *T. bidentata* from the Pacific Ocean.

The STRUCTURE analysis indicated that the most likely number of genetic clusters was *K* = 4, thus supporting the four clades obtained with the phylogenetic analyses (Figure 3a,b and Figure S1). The STRUCTURE analysis further revealed that there is no admixture between the Indian Ocean and the other clusters and very little admixture between the Marquesas and the rest of the Pacific. However, STRUCTURE did detect a gradient of admixture from the West Pacific cluster into the Central Pacific cluster. Sub‐structure was only detected in the Indian Ocean (*K* = 2; Figure 3a,b and Figure S1), where two genetic clusters emerged, Red Sea and Southwest Indian Ocean. The same four main genetic clusters and patterns of admixture between West and Central Pacific were also recovered with the PCA, Pairwise *F*_ST_, and AMOVA analyses (Figure 4 and Table 3). Similar results were obtained with the SNPs under selection (Figure S2).

**TABLE 3:** Pairwise *F*_ST_ calculated between the four genetic clusters (on top) and hierarchical AMOVA between the four genetic clusters (below).

Pairwise *F*_ST_	Indian Ocean	Marquesas Is.	West Pacific	Central Pacific
Indian Ocean	—			
Marquesas Is.	**0.25**	—		
West Pacific	**0.27**	**0.1**	—	
Central Pacific	**0.29**	**0.09**	**0.04**	—
**AMOVA**	***%***	**Obs**.	**Std.Obs**.	***p‐value***
Within samples	76.71	954.05	−12.08	0.01
Between samples	9.66	120.13	6.74	0.01
Between genetic clusters	13.63	169.54	49.57	0.01

*Note:* Significant values are indicated in bold (*p*‐value < 0.001).

Pairwise *F*_ST_ estimated between clusters were all significant (Table 3). As expected, the highest pairwise *F*_ST_ values were recovered between the Indian Ocean and all others (average 0.27 ± 0.02). The lowest genetic distances were recovered between the West and Central Pacific clusters (0.04), supporting the pattern obtained with the STRUCTURE analyses. Despite the Central Pacific cluster containing all other Polynesian samples, the Marquesas cluster showed equal pairwise *F*_ST_ values with the West and Central Pacific clades (0.1 and 0.09, respectively).

The hierarchical AMOVA showed that there was significant variation between genetic clusters, individuals, and alleles (Table 3). Most of the variation resides within samples (or between alleles). However, a significant amount of variation was also found between genetic clusters (13.63%). IBD was detected within the Pacific populations (*p*‐value = 0.04, Figure S3).

Genetic diversity analyses revealed that all genetic clusters had similar observed and expected heterozygosity, average nucleotide diversity, and *F*_IS_, except for the West Pacific clade that had noticeably lower diversity and *F*_IS_ values. The Indian Ocean cluster had the lowest proportion of polymorphic sites (0.67), while the West Pacific the highest (0.98) (Table 4).

**TABLE 4:** Population genetic summary statistics calculated for each of the four genetic clusters. Proportion of polymorphic sites is calculated from the fraction of variant/total sites.

Genetic cluster	# of samples	Proportion of polymorphic sites	Observed heterozygosity (*H*_O_)	Expected heterozygosity (*H*_S_)	Inbreeding coefficient (*F*_IS_)
Indian Ocean	11	0.67	0.29	0.34	0.16
Marquesas Is.	11	0.87	0.28	0.33	0.15
Central Pacific	25	0.94	0.24	0.28	0.15
West Pacific	72	0.98	0.23	0.26	0.12

### 3.4 Morphometrics

**FIGURE 5:**
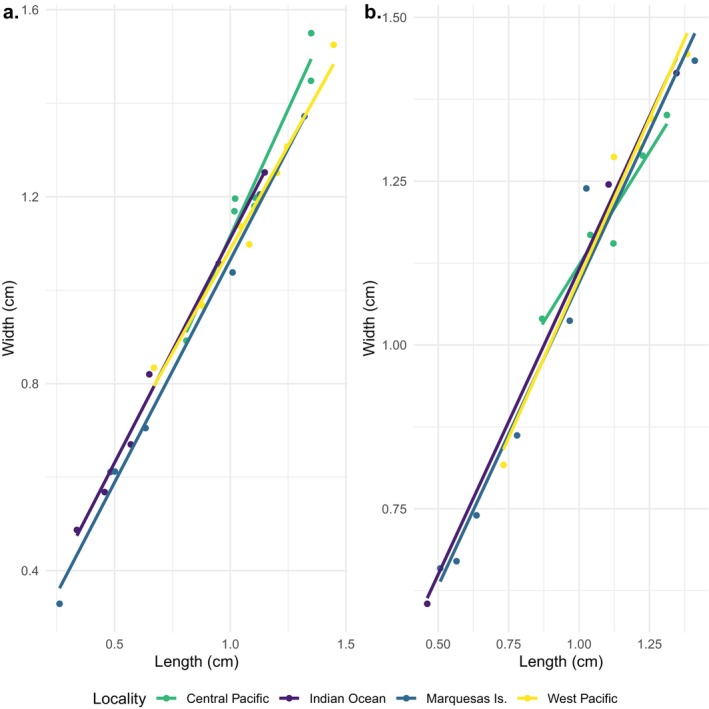
Length over width of carapace in centimeters for (a) male and (b) female crabs in the four genetic clusters.

None of the observed variations in carapace size between the four genetic clusters were significant (*p*‐value = 0.07 and 0.441 for males and females, respectively). Similarly, measured carapace differences between sexes were also not significant (*p*‐value = 0.589, Figure 5).

## 4 Discussion

Because they protect their host from predators and harmful sediment deposits, coral guard crabs play an important role in coral health and survival (Glynn 1980; Pratchett 2001; Shima et al. 2010; Stier et al. 2010; Stewart et al. 2006; Stier and Osenberg 2024). Obtaining tissue samples from multiple species or populations of *Trapezia* from multiple localities remains a difficult task that has limited phylogenetics and population genomics studies of this group. As a result, and despite their ecological importance, little is known about the actual status of the genus and the population dynamics of some of the most common and widespread species. Here, we show that a GRAS‐Di approach with both fresh and museum samples can help untangle the phylogeny of this genus (although still incomplete), identify potential cryptic species, and improve our understanding of the ecological and historical drivers of genetic divergence of a widespread species of *Trapezia* crab. The use of a PCR‐based library preparation approach allowed us to obtain high yields of SNP from low concentration and degraded museum DNA. There was no significant correlation between DNA quality and yielded SNP. Additionally, the amount and quality of the data was equal to or superior to what can commonly be obtained from (dd)RAD‐seq or other genome sub‐sampling approaches on fresh samples. This allowed us to implement stringent data filtering, retaining only SNPs present in 50% of samples for our analysis, affirming the efficacy of our approach to study the evolutionary history of marine invertebrate taxa with archival museum DNA.

### 4.1 A Clarified *Trapezia* Phylogeny

Our phylogenetic analyses, conducted with 164 Trapezia individuals and 11,479 SNPs, allowed us to clarify the phylogenetic relationships of 12 species within the genus *Trapezia*. Significantly, we found that *T. rufopunctata* and *T. flavopunctata* are the most genetically distant species within the *Trapezia* genus. *Trapezia rufopunctata* and *T. flavopunctata* are the largest species of the genus distributed across the Indo‐Pacific and usually found in larger coral species such as *Pocillopora eydouxi* . Their large size confers them the ability to efficiently defend their coral host against large corallivorous predators such as the crown of thorn seastar *Acanthaster plancii* (McKeon and Moore 2014). The position of these two species at the tip of a long branch had been previously recovered (Canizales‐Flores et al. 2020; McKeon 2010 unpublished work) and prompted the hypothesis that they might constitute a separate genus entirely. The topology of our tree tends to support this hypothesis; however, more complete phylogenetic analyses with all 22 currently recognized *Trapezia* species and a few outgroup species, accompanied with morphometric analyses, are necessary to confirm it (Castro et al. 2004). Our analyses placed *T. lutea* and *T. cymodoce* as sister species, agreeing with Rouzé et al. (2017)'s 16S and COI trees, and clarified the ambiguous positions of all other species. Interestingly, we recovered five *T. tigrina* individuals from Guam, a locality in which their presence had not been recorded previously (Castro et al. 2004; metadata and live photo available through the University of Guam biorepository online: http://specifyportal.uog.edu/; catalog numbers can be found in Table S1).

### 4.2 Past Geographic Barriers Promote Cryptic Speciation in the Widespread *T. bidentata*

Among the 22 currently recognized *Trapezia* species, merely four, *T. bidentata* included, exhibit a broad distribution throughout the Indo‐Pacific, an observation that potentially correlates with their pelagic larval duration (Castro et al. 2004). Although the exact length of *T. bidentata* 's pelagic larval phase is unknown, marine crab larvae typically spend between 5 weeks to 4 months as plankton (Shikatani and Shokita 1990; Effendy et al. 2021; Mohamedeen and Hartnoll 1989; Moloney et al. 1994). Within *T. bidentata* , we found evidence of four distinct genetic clusters: the Indian Ocean (Saudi Arabia, Madagascar, Mayotte, and Mascarene Islands), West Pacific (Mariana Islands, Marshall Islands, North Line islands, Phoenix islands, and Papua New Guinea), Central Pacific (French Polynesia, American Samoa, and South Line Islands), and the Marquesas Islands.

The greatest genetic divergence within *T. bidentata* is found between the Indian and Pacific Oceans. The genetic separation between the two ocean basins often dates back to the Last Glacial Maximum (LGM), during which sea level dropped, exposing several shallow land masses and effectively isolating the two ocean basins from one another. Two of these land masses, the Sunda Shelf, a shallow reservoir connecting Southeast Asian countries, and the Torres Strait, a land bridge connecting Northeastern Australia and Papua New Guinea, reduced flow between the Indian and Pacific Oceans, creating a formidable barrier to marine larval dispersal between the two basins (Voris 2000). This temporary restriction of gene flow between the oceans led to several genetic divergences between marine organisms including populations of *Tridacna* clams, the false clownfish *Amphiprion ocellaris* , and the brooding coral *Seriatopora hystrix* (Van Der Ven et al. 2021; Keyse et al. 2018; DeBoer et al. 2014; Nelson and Chan 2000). The strong genetic divergence between the Indian and Pacific Ocean populations of *T. bidentata* , supported by our STRUCTURE analysis, PCA, and pairwise *F*_st_ values, strongly suggests that *T. bidentata* was also impacted by these historical barriers. Further, the complete lack of admixture and the fact that genetic distances between the Indian Ocean populations and all other Pacific populations are as high or higher than distances observed between three groups of recognized *Trapezia* sister species ( *T. rufopunctata* and *T. flavopunctata* , *T. serenei* and *T. tigrina* , *T. guttata* and *T. punctimanus*) strongly suggest cryptic allopatric speciation. Interestingly, variation in color patterns between these geographically separated groups were identified in an unpublished work by McKeon (2010). While we cannot corroborate McKeon's (2010) results without examining fresh specimens, prior observations and our genetic evaluation of the species allow us to speculate that *T. bidentata* from the Indian Ocean is in fact a separate sister species.

Our data also reports limited connectivity between Red Sea and southern Indian Ocean populations as represented in our phylogenetic tree, PCA, and STRUCTURE analyses (Figures 3 and 4). Barriers to dispersal between the Red Sea and the rest of the Indian Ocean can be attributed to local eddies within the Arabian Sea likely impeding larval transport to the east, and seasonal interactions between the South Equatorial Current, the Somali Current, and the Equatorial Countercurrent isolating northern from southern populations (Farhadi et al. 2017; Fauvelot et al. 2020). These oceanographic barriers are, for example, responsible for the limited connectivity between spiny lobster ( *Panulirus homarus* ) populations in the northern Indian Ocean, despite having a long pelagic larval phase upward of 6 months (Farhadi et al. 2017). Further, seasonal cold‐water upwelling events may impede the dispersal and survival of sensitive larvae along with the high levels of salinity and temperature that are found in the Red Sea (Torquato et al. 2019; DiBattista et al. 2016).

### 4.3 Genetic Isolation of the Marquesas Islands

The Marquesas Islands, despite their proximity to the Central Pacific cluster, were genetically distinct from neighboring populations, as displayed in all our analyses. Although thus far undetected in *T. bidentata* , this divergence is concordant with extensive reports of population genetic divergence and high levels of endemism within the Marquesas Islands (Randall 2001; Gaither et al. 2010; Szabó et al. 2014). Genetic divergence and endemism in the Marquesas Islands are thought to be driven by the Marquesas Counter Current flowing in opposite direction of the South Equatorial Current, creating a barrier to dispersal between the Marquesas and their nearest neighboring islands of the Tuamotu Archipelago (500 km South) as well as by equatorial upwelling ENSO events causing unique sea temperatures in the archipelago (Randall 2001; Lemer et al. 2014).

While most studies regarding endemism at this locality widely focus on reef fish populations, a small collection of studies speak of genetic divergence in species with a long pelagic dispersal phase such as the black‐lipped pearl oyster *Pinctata margaritifera* (15–30‐day dispersal; Thomas et al. 2014; Lemer et al. 2014; Reisser et al. 2019). Here, we identified minimal admixture between the Marquesas Islands and any other population. It is thus likely that, despite its putative long pelagic larval phase, *T. bidentata* is also strongly affected by the various environmental factors limiting genetic connectivity in the archipelago. Further, reef systems throughout the Marquesas archipelago are patchy, scattered, and low in coral diversity and lack any form of barrier reef (Randall 2001; Fey et al. 2020). The combination of harsh currents and the lack of protection in the form of a barrier reef may create a strong barrier to dispersal for *T. bidentata* , and may potentially promote speciation (Lal et al. 2017; Lemer et al. 2014; Thomas et al. 2014). We hypothesize that the Marquesas clade of *T. bidentata* is most likely a different species, potentially aligning with Hombron and Jacquinot's original findings of *T. miniata* in 1846. However, the most distinctive feature of *T. miniata* compared to *T. bidentata* is the lack of a red dot on its terminal ambulatory leg and it remains difficult to conclude such without fresh specimens (Castro et al. 2004; 1996; Hombron and Jacquinot 1842). Therefore, we encourage morphological analyses with fresh samples to confirm the hypothesis that this putative Marquesas endemic is a jr. synonym of *T. bidentata*.

### 4.4 High Connectivity Throughout the Pacific Ocean

Marquesas Islands aside, *T. bidentata* in the Pacific Ocean form two intermixed genetic groups: the Central and West Pacific. While other genetic clusters within our study can be explained by historical, oceanographic, or biophysical barriers, the incomplete delineation between the Central and West Pacific in *T. bidentata* is likely attributed to geographic distance, habitat heterogeneity, and current patterns via oceanic gyres. While most currents stimulate population connectivity across the Pacific Ocean, regional circulation patterns and extensive geographic distances along with varying habitat geomorphology help maintain some population structure throughout the ocean basin, especially between the most distant islands (Lal et al. 2017). However, the North and South oceanic gyres, which have caused divergence between the North and South Pacific in other taxa, could explain the clear genetic split between the North and South Line islands and between the Phoenix islands and American Samoa (Figure 3; Goetze 2005; Norton and Goetze 2013). As our sampling sites within the Pacific Ocean cover an expanse of 9,100 km between our two furthest sites (Mariana Islands and Tuamotu Archipelago), it is reasonable to expect that mixing between these distant populations is infrequent. However, considering the putative long planktonic larval phase of *T. bidentata* and the numerous islands present in the Central Pacific, a stepping‐stone model could explain larval dispersal throughout the entire Pacific Ocean, with time, connecting the most distant islands. For instance, the Micronesian islands are theorized to function as a series of stepping stones connecting populations of the scleractinian corals *Acropora hyacinthus* and *A. digitifera* from the Coral triangle and the Central pacific, following an IBD model of dispersal (Davies et al. 2015). Since we detected IBD in our data (Figure S3), it is likely that distribution of *T. bidentata* throughout the West and Central Pacific follows this model. The distinct genetic signatures observed in the two clusters are potentially due to the directionality of equatorial currents pushing larvae away from the equator, resulting in higher retention of larvae within each genetic cluster (Scheltema 1994).

It is important to note that specimens from Hawaii and the East Pacific have not been included in the present study despite the range of *T. bidentata* expanding as far. Hawaii is the most geographically isolated archipelago, separated from its nearest neighbors, the Marshall Islands, and larger landmasses such as the East Coast of North America, by a vast expanse of open ocean. This isolation combined with its distance from the Coral Triangle has resulted in high levels of genetic differentiation and endemism among Hawaiian marine fauna, highlighting a distinctive evolutionary pathway (Kay and Palumbi 1987; Bhattacharya et al. 2022). The isolation of the Eastern Pacific from the rest of the Pacific Ocean, combined with distinct environmental conditions such as upwellings, similarly drives increased genetic divergence with West and Central Pacific regions (Baums et al. 2012; Chow et al. 2011). While it is impossible to determine how the inclusion of Trapezia specimens from Hawaii or the Eastern Pacific might have influenced our results, it is likely that these populations would exhibit significant genetic differentiation from those in the West and Central Pacific, as suggested by Huber (1985) and other studies on pelagic‐dispersing organisms (Bowen 2016; Kay and Palumbi 1987).

## 5 Conclusion

Overall, the geographic span covered by *T. bidentata* in the Pacific clusters suggests that *T. bidentata* has an extensive pelagic larval phase, allowing it to travel great distances and mix with neighboring populations. This ability of an otherwise sedentary organism to disperse throughout the Pacific with little impedance can lead to an expansive gene pool, with the potential to greatly increase adaptive capacity. High adaptive capacity is essential for the survival of species in the context of climate change as reef health is affected by regional influences such as storms, bleaching events, and anthropogenic impacts, among other factors (Bruno and Selig 2007). However, considering the mutualistic relationship of *Trapezia* crabs with Pocilloporid corals, their ability to colonize new habitats is dependent on host availability and as such they remain highly threatened by the changing climate and rising sea temperatures. The presence of putative distinct species in the Indian Ocean and the Marquesas Islands strongly indicates that the greatest drivers of divergence in this taxon are currents and historic physical barriers to dispersal, and that allopatry is the primary method of speciation within *Trapezia*. It is thus important to investigate and demystify other supposedly widespread species within this genus and to increase the number of genetic studies in this system. As species and populations disappear at an unparalleled pace (Raven and Miller 2020), the need to accurately characterize global biodiversity is growing and museum‐based approaches like the one implemented here can help with this task.

## Author Contributions

**Kenzie Pollard:** conceptualization (equal), data curation (lead), formal analysis (lead), methodology (equal), writing – original draft (lead), writing – review and editing (equal). **Carlos Leiva:** data curation (equal), formal analysis (equal), methodology (supporting), writing – review and editing (supporting). **Heloise Rouzé:** data curation (supporting), methodology (supporting), writing – review and editing (supporting). **Sarah Lemer:** conceptualization (lead), data curation (equal), formal analysis (equal), funding acquisition (lead), methodology (equal), supervision (lead), writing – original draft (equal), writing – review and editing (lead).

## Conflicts of Interest

The authors declare no conflicts of interest.

## Supporting information


**Figure S1.** Evanno Method results for choosing *K* for STRUCTURE plots of (a) all of *T. bidentata* (*K* = 4) and (b) within the Indian Ocean cluster (*K* = 2). From top left, plots measure mean estimated ln probability of data with standard error, the first derivative with standard error, the absolute value of the second derivative with standard error, and delta *K*.

**Figure S2.** (a) STRUCTURE and (b) principal component analysis (PCA) analyses of single nucleotide polymorphism (SNPs) under selection of all *T. bidentata* samples.

**Figure S3.** Isolation by distance analysis of Pacific populations. The outer and inner colors indicate the pairwise comparisons between populations. The gray area around the line indicates standard error.

**Figure S4.** Matrix representing sample coverage. Black = locus present, white = locus absent. Blue box highlights *T. rufopunctata* and *T. flavopunctata*. Source code: de Medeiros BA. (2019) Matrix Condenser v.1.0. Available at: https://github.com/brunoasm/matrix_condenser/.

**Table S1.** List of samples. Note that not all samples from the museums were correctly identified (indicated in “Note”). As many samples were listed under the same accession numbers, the column “# specimens per lot” indicates the number of samples under the same accession number. Fresh samples collected in Guam are listed as their sample ID rather than accession number.

**Table S2.** Table of sample DNA concentration, coverage, and retained reads (as presented in Figure 1). Information about whether samples are from museum or freshly sampled and whether the sample was filtered out of the analyses is indicated.

## Data Availability

The raw reads and SNP matrices can be found here: https://doi.org/10.5061/dryad.x0k6djhtr. Scripts are available for download here: https://github.com/kenziepollard/trapezia and via Dryad (https://doi.org/10.5061/dryad.x0k6djhtr).
