# Supplementary figures and images for: Genomic Insights From Natural History Collections Reveal Cryptic Speciation in Coral Guard Crabs (Family: Trapeziidae)
Source: Ecol Evol. 2025 Feb 19;15(2):e70960. doi: 10.1002/ece3.70960 (PMC11836902; doi:10.1002/ece3.70960)

a.

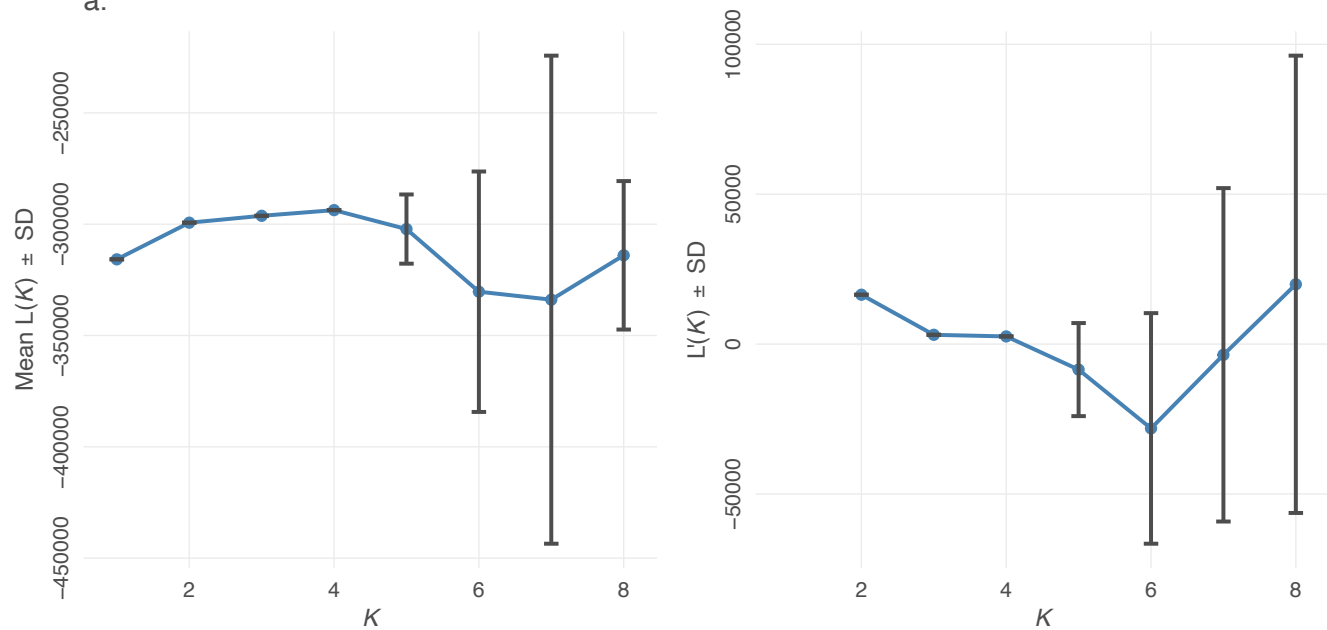

b.

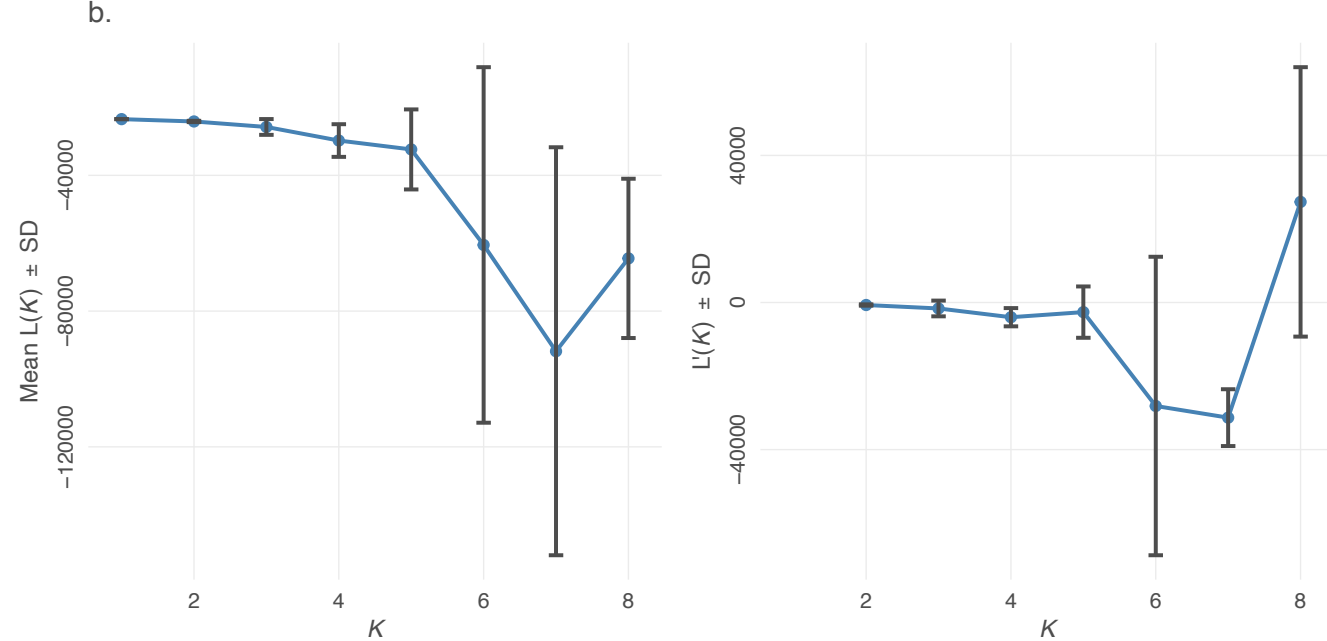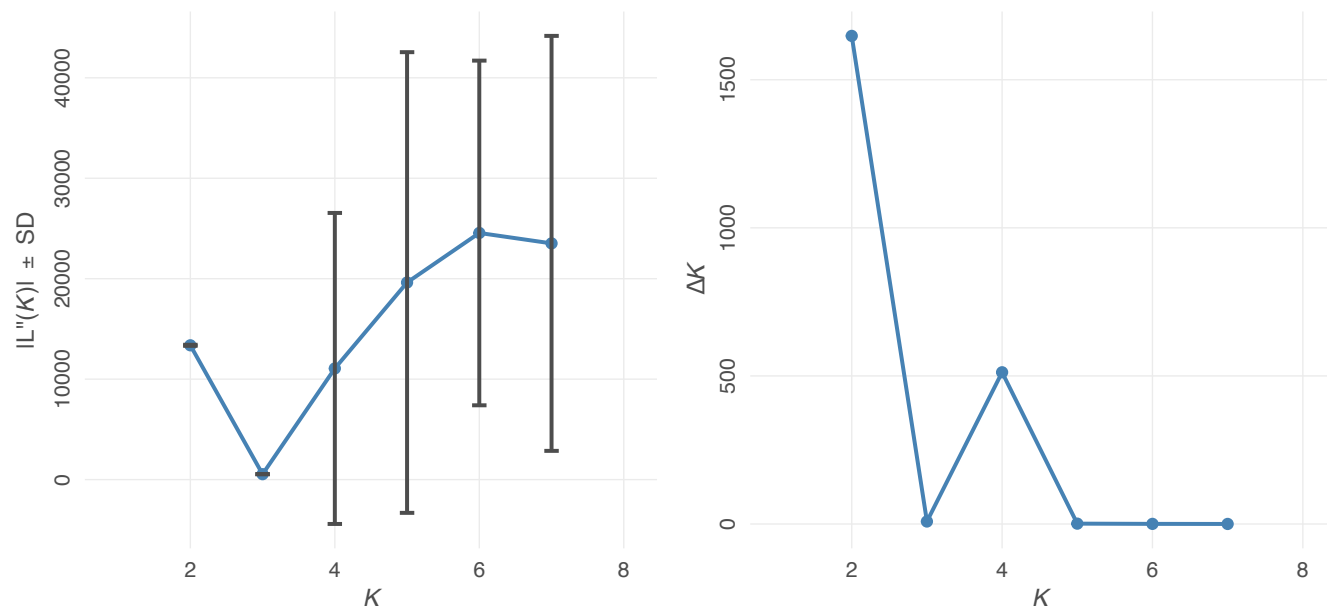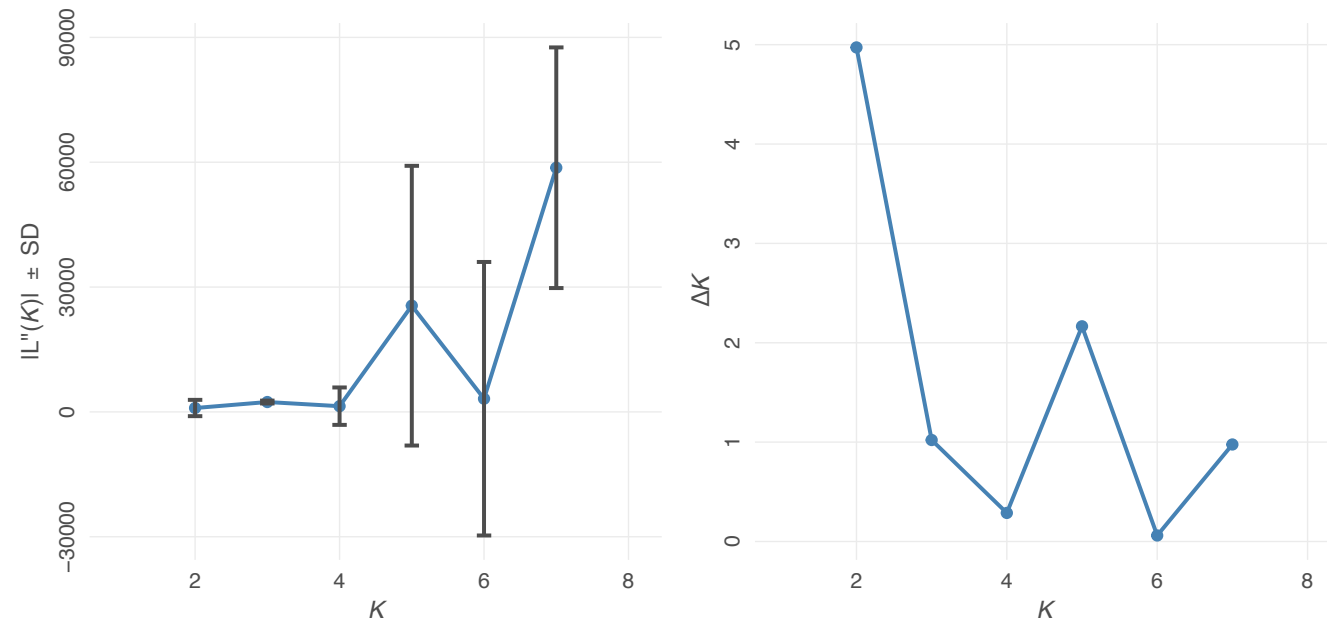

Supplement: Supplementary file 1 — Figure S1. Evanno Method results for choosing K for STRUCTURE plots of (a) all of T. bidentata (K = 4) and (b) within the Indian Ocean cluster (K = 2). From top left, plots measure mean estimated ln probability of data with standard error, the first derivative with standard error, the absolute value of the second derivative with standard error, and delta K. [file ECE3-15-e70960-s004.pdf]

a.

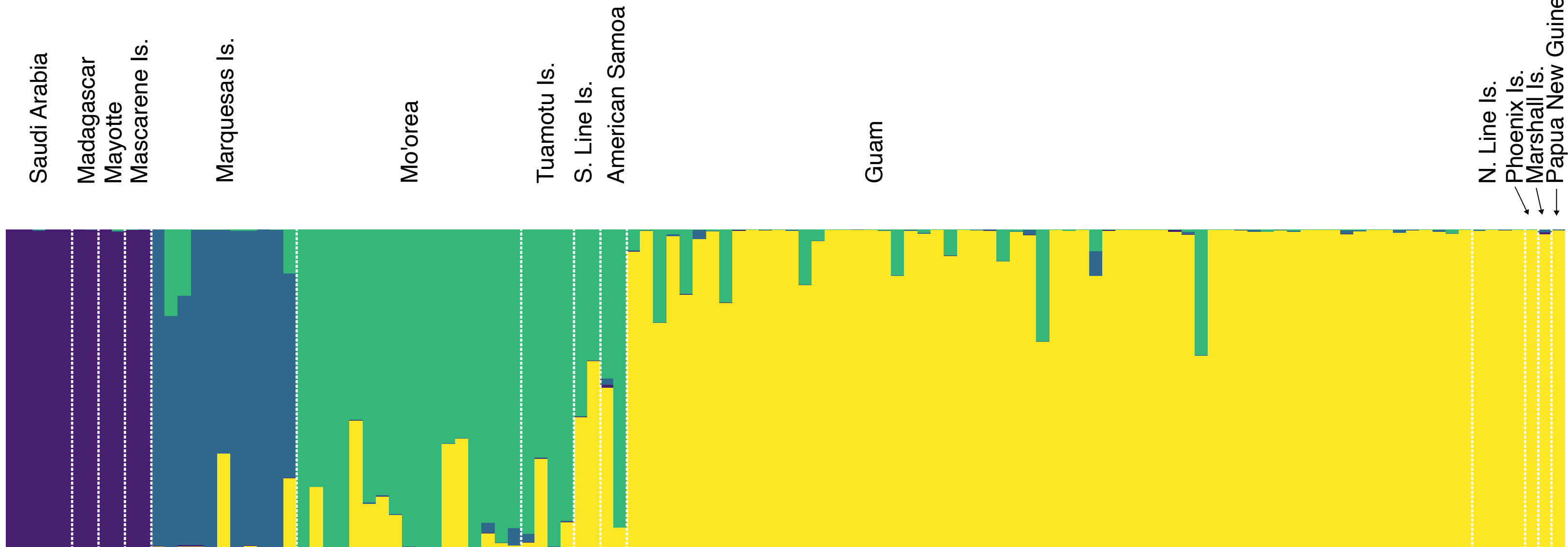

b.

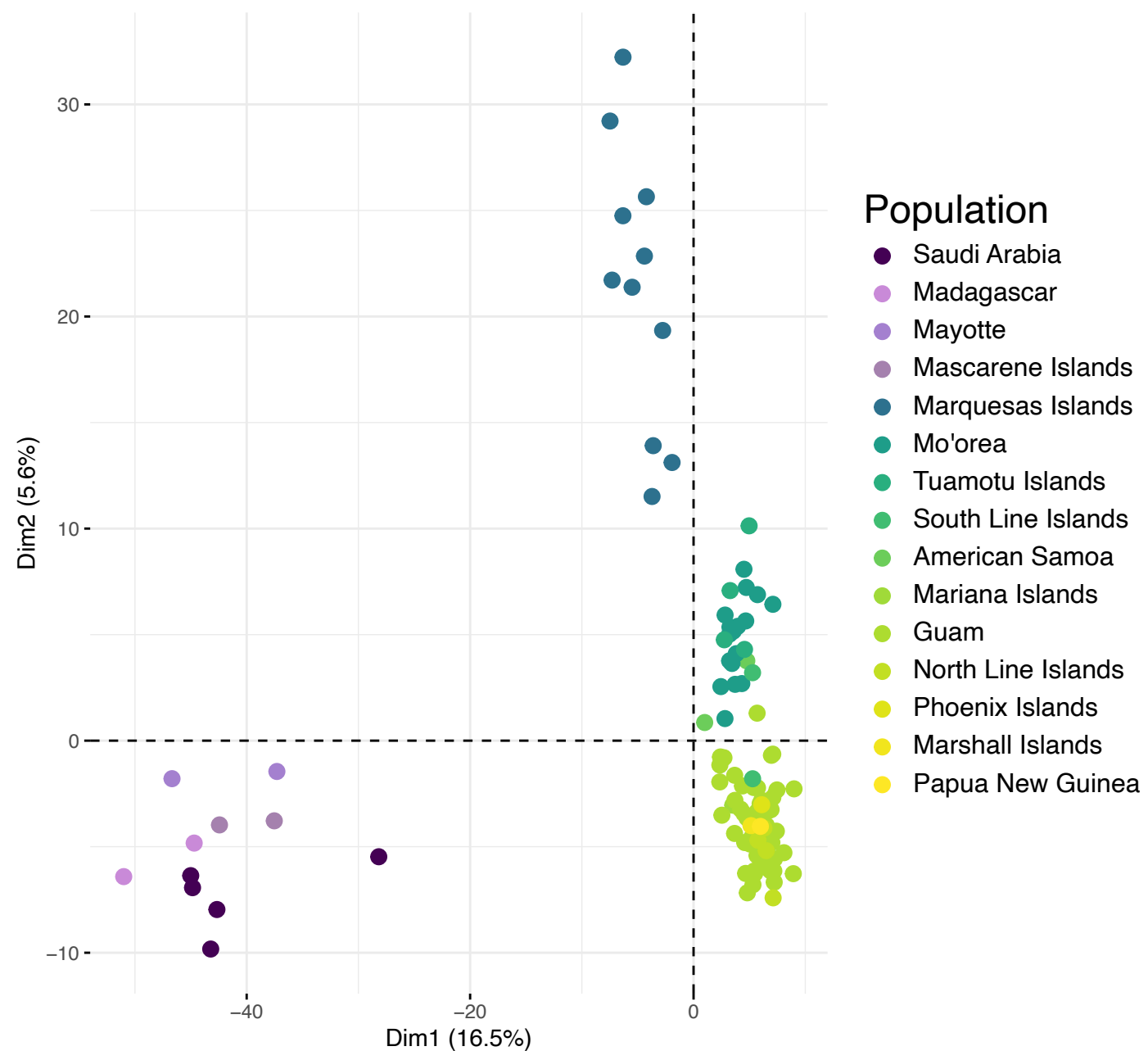

Supplement: Supplementary file 2 — Figure S2. (a) STRUCTURE and (b) principal component analysis (PCA) analyses of single nucleotide polymorphism (SNPs) under selection of all T. bidentata samples. [file ECE3-15-e70960-s005.pdf]

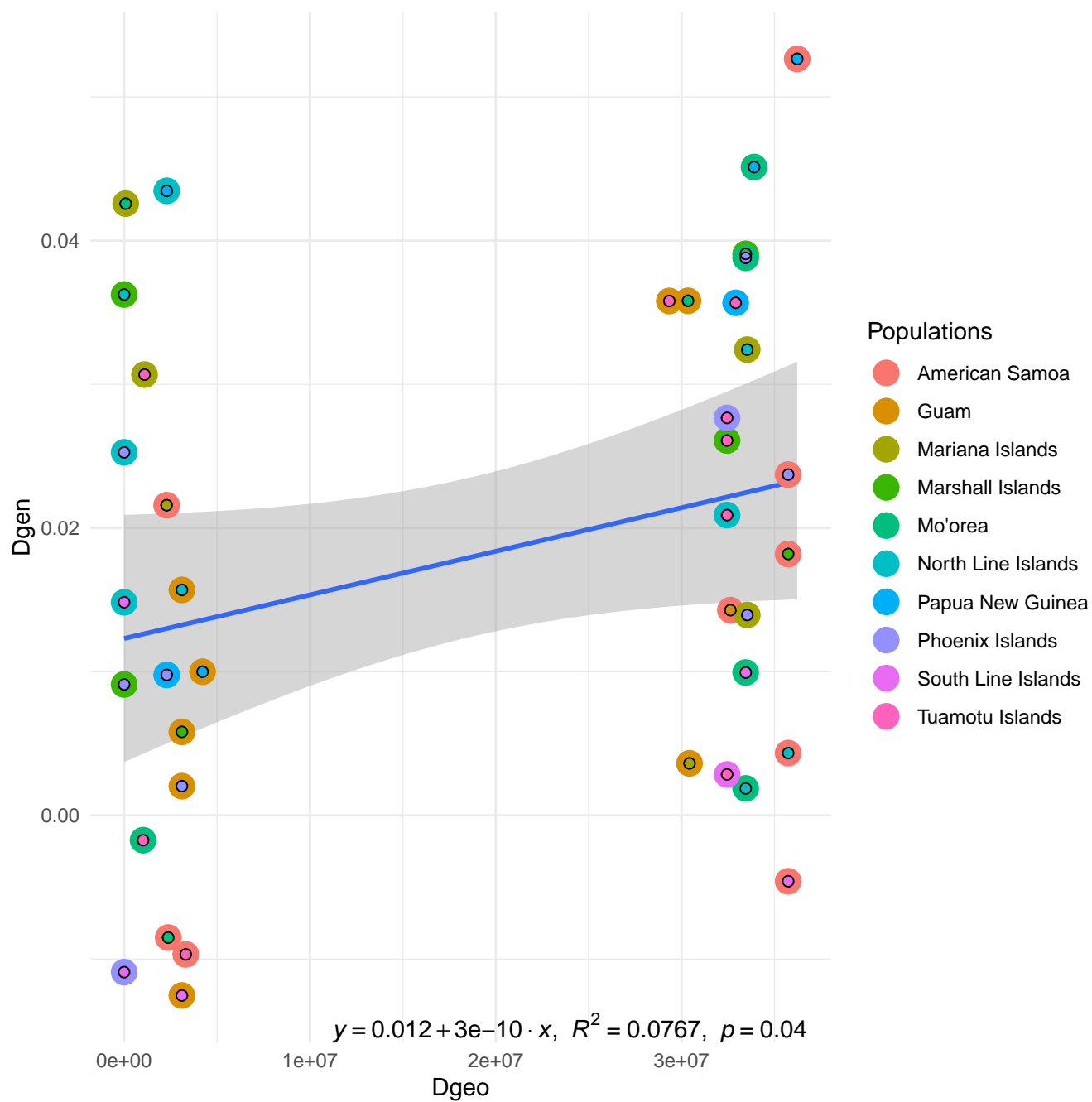

Supplement: Supplementary file 3 — Figure S3. Isolation by distance analysis of Pacific populations. The outer and inner colors indicate the pairwise comparisons between populations. The gray area around the line indicates standard error. [file ECE3-15-e70960-s002.pdf]
