# Supplementary material for: Genomic Insights From Natural History Collections Reveal Cryptic Speciation in Coral Guard Crabs (Family: Trapeziidae)
Source: Ecol Evol. 2025 Feb 19;15(2):e70960. doi: 10.1002/ece3.70960 (PMC11836902; doi:10.1002/ece3.70960)
Supplement: Supplementary file 4 — Figure S4. Matrix representing sample coverage. Black = locus present, white = locus absent. Blue box highlights T. rufopunctata and T. flavopunctata. Source code: de Medeiros BA. (2019) Matrix Condenser v.1.0. Available at: https://github.com/brunoasm/matrix_condenser/. [file ECE3-15-e70960-s006.pdf]

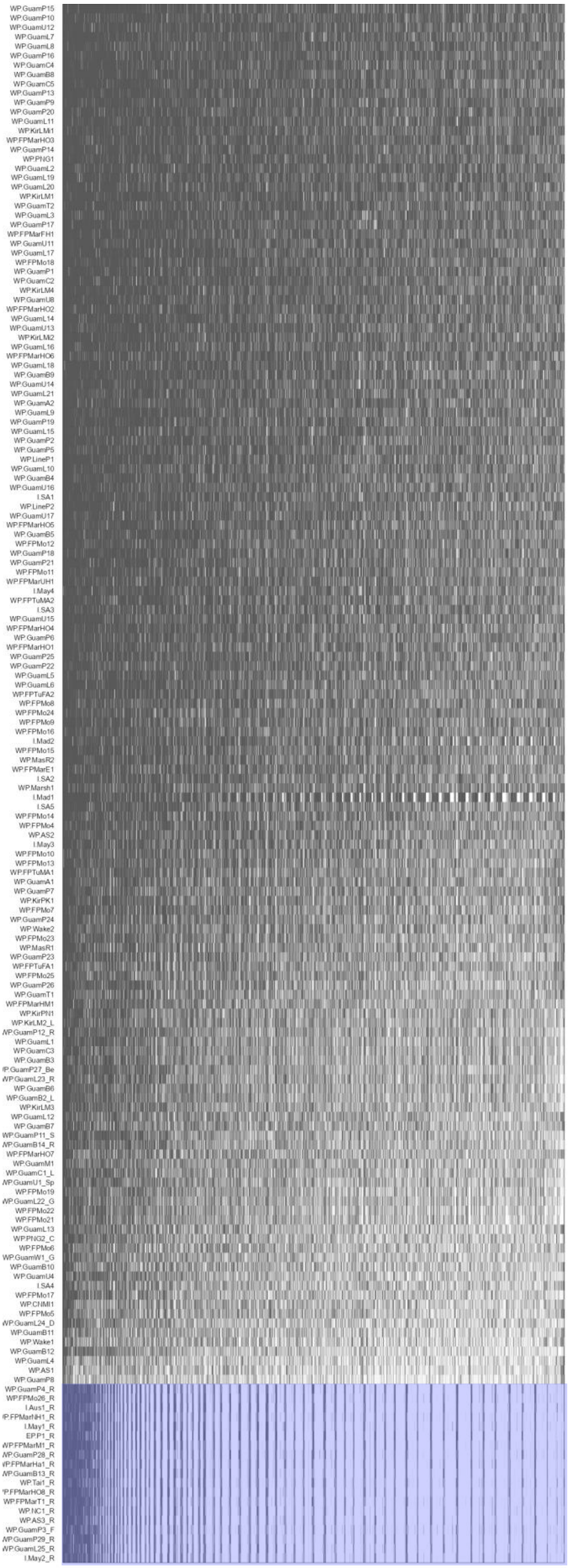
WP.GuamP15
WP.GuamP10
WP.GuamU12
WP.GuamL7
WP.GuamL8
WP.GuamP16
WP.GuamC4
WP.GuamB8
WP.GuamC5
WP.GuamP13
WP.GuamP9
WP.GuamP20
WP.GuamL11
WP.KirLMi1
WP.FPMarHO3
WP.GuamP14
WP.PNG1
WP.GuamL2
WP.GuamL19
WP.GuamL20
WP.KirLM1
WP.GuamT2
WP.GuamL3
WP.GuamP17
WP.FPMarFH1
WP.GuamU11
WP.GuamL17
WP.FPMo18
WP.GuamP1
WP.GuamC2
WP.KirLM4
WP.GuamU8
WP.FPMarHO2
WP.GuamL14
WP.GuamU13
WP.KirLMi2
WP.GuamL16
WP.FPMarHO6
WP.GuamL18
WP.GuamB9
WP.GuamU14
WP.GuamL21
WP.GuamA2
WP.GuamL9
WP.GuamP19
WP.GuamL15
WP.GuamP2
WP.GuamP5
WP.LineP1
WP.GuamL10
WP.GuamB4
WP.GuamU16
I.SA1
WP.LineP2
WP.GuamU17
WP.FPMarHO5
WP.GuamB5
WP.FPMo12
WP.GuamP18
WP.GuamP21
WP.FPMo11
WP.FPMarUH1
I.May4
WP.FPTuMA2
I.SA3
WP.GuamU15
WP.FPMarHO4
WP.GuamP6
WP.FPMarHO1
WP.GuamP25
WP.GuamP22
WP.GuamL5
WP.GuamL6
WP.FPTuFA2
WP.FPMo8
WP.FPMo24
WP.FPMo9
WP.FPMo16
I.Mad2
WP.FPMo15
WP.MasR2
WP.FPMarE1
I.SA2
WP.Marsh1
I.Mad1
I.SA5
WP.FPMo14
WP.FPMo4
WP.AS2
I.May3
WP.FPMo10
WP.FPMo13
WP.FPTuMA1
WP.GuamA1
WP.GuamP7
WP.KirPK1
WP.FPMo7
WP.GuamP24
WP.Wake2
WP.FPMo23
WP.MasR1
WP.GuamP23
WP.FPTuFA1
WP.FPMo25
WP.GuamP26
WP.GuamT1
WP.FPMarHM1
WP.KirPN1
WP.KirLM2_L
WP.GuamP12_R
WP.GuamL1
WP.GuamC3
WP.GuamB3
P.GuamP27_Be
WP.GuamL23_R
WP.GuamB6
WP.GuamB2_L
WP.KirLM3
WP.GuamL12
WP.GuamB7
WP.GuamP11_S
WP.GuamB14_R
WP.FPMarHO7
WP.GuamM1
WP.GuamC1_L
WP.GuamU1_Sp
WP.FPMo19
WP.GuamL22_G
WP.FPMo22
WP.FPMo21
WP.GuamL13
WP.PNG2_C
WP.FPMo6
WP.GuamW1_G
WP.GuamB10
WP.GuamU4
I.SA4
WP.FPMo17
WP.CNMI1
WP.FPMo5
WP.GuamL24_D
WP.GuamB11
WP.Wake1
WP.GuamB12
WP.GuamL4
WP.AS1
WP.GuamP8
WP.GuamP4_R
WP.FPMo26_R
I.Aus1_R
P.FPMarNH1_R
I.May1_R
EP.P1_R
WP.FPMarM1_R
WP.GuamP28_R
WP.FPMarHa1_R
WP.GuamB13_R
WP.Tai1_R
P.FPMarHO8_R
WP.FPMarT1_R
WP.NC1_R
WP.AS3_R
WP.GuamP3_F
WP.GuamP29_R
WP.GuamL25_R
I.May2_R
